# Highly Water-Dispersed Natural Fullerenes Coated with Pluronic Polymers as Novel Nanoantioxidants for Enhanced Antioxidant Activity

**DOI:** 10.3390/antiox13101240

**Published:** 2024-10-15

**Authors:** Hyeryeon Oh, Jin Sil Lee, Panmo Son, Jooyoung Sim, Min Hee Park, Young Eun Bang, Daekyung Sung, Jong-Min Lim, Won Il Choi

**Affiliations:** 1 Center for Bio-Healthcare Materials, Bio-Convergence Materials R&D Division, Korea Institute of Ceramic Engineering and Technology, 202, Osongsaengmyeong 1-ro, Osong-eup, Heungdeok-gu, Cheongju 28160, Republic of Korea; hyeryeon.oh@kicet.re.kr (H.O.); jslee92@kicet.re.kr (J.S.L.); spm1006@kicet.re.kr (P.S.); jynjc1110@naver.com (J.S.); conomo0850@gmail.com (M.H.P.); dksung@kicet.re.kr (D.S.); 2 School of Materials Science and Engineering, Gwangju Institute of Science and Technology, 123, Cheomdan-gwagiro, Buk-gu, Gwangju 61005, Republic of Korea; 3 Department of Applied Bioengineering, Graduate School of Convergence Science and Technology, Seoul National University, Seoul 08826, Republic of Korea; 4 Department of Electronic Materials, Devices, and Equipment Engineering, Soonchunhyang University, 22 Soonchunhyang-ro, Shinchang-myeon, Asan-si 31538, Republic of Korea; dktks17@naver.com; 5 Department of Chemical Engineering, Soonchunhyang University, 22 Soonchunhyang-ro, Shinchang-myeon, Asan-si 31538, Republic of Korea

**Keywords:** fullerene, Pluronic, colloidal stability, antioxidant, ROS scavenging

## Abstract

Fullerene is a cosmic material with a buckyball-like structure comprising 60 carbon atoms. It has attracted significant interest because of its outstanding antioxidant, antiviral, and antibacterial properties. Natural fullerene (NC60) in shungite meets the demand of biomedical fields to scavenge reactive oxygen species in many diseases. However, its hydrophobicity and poor solubility in water hinder its use as an antioxidant. In this study, highly water-dispersed and stable Pluronic-coated natural fullerene nanoaggregates (NC60/Plu) were prepared from various Pluronic polymers. The water dispersity and stability of NC60 were compared and optimized based on the characteristics of Pluronic polymers including F68, F127, L35, P123, and L81. In particular, NC60 coated with Pluronic F127 at a weight ratio of 1 to 5 showed excellent antioxidant effects both in situ and in vitro. This suggests that the high solubilization of NC60 in Pluronic polymers increases its chance of interacting with reactive oxygen radicals and improves radical scavenging activity. Thus, the optimized NC60/PF127 may be a novel biocompatible antioxidant for treating various diseases associated with oxidative stress.

## 1. Introduction

Fullerene (C60), the third carbon allotrope, was first found in the gas emitted by a dying star in space. Its discovery has attracted significant interest from researchers because of its buckyball-like structure, which provides unique physicochemical, electronic, optical, and reactive properties [1,2]. Therefore, many attempts have been made to develop C60 in the laboratory, facilitating its application in drug delivery systems [3], nanoelectronics [4], sensors [5], fuel storage [6], catalysts [7], and environmental remediation [8]. R. F. Curl, H. W. Kroto, and R. E. Smalley received the Nobel Prize in Chemistry for discovering C60. They found that vaporization of graphite using laser irradiation produced a carbon cluster composed of 60 carbon atoms [9]. However, it is not suitable for industrial applications owing to its high cost and difficult synthesis. Another method of C60 synthesis is the extraction of naturally occurring C60 present in carbon-containing rock shungite [10]. Reznikov et al. suggested the possible application of natural fullerene (NC60) extracted from shungite in the medical and ecological fields [11]. The NC60 was found to be particularly intriguing for researchers due to the expectation of greener and more sustainable methods for its production.

The NC60 possesses excellent antioxidant, antiviral, and antibacterial activities [12,13]. In particular, it is often named “radical sponges” as it shows a high reactivity towards the reactive oxygen species (ROS) owing to delocalized π-electrons over its carbon core [14]. ROS are highly reactive oxygen derivatives and mainly include superoxide (O_2_^•−^), hydroxyl radicals, hydrogen peroxide, and hypochlorite. Its 30 carbon double bonds readily entrap and inactivate these free radicals [15]. It has been reported that the NC60 can scavenge superoxide, hydroxyl, and lipid radicals [15,16,17]. In living organisms, the antioxidant defense system closely monitors and controls the balance between the formation and neutralization of ROS. They can protect the body against harmful pathogens and participate in physiological activities including wound healing and tissue regeneration. However, the abnormal overexpression of ROS results in oxidative stress, which can cause tissue damage and contribute to various diseases such as atherosclerosis, inflammation, and tumor [18,19,20]. Thus, the NC60 may be an outstanding antioxidant for treatment of ROS-mediated diseases. However, its use in biomedical applications remains limited because of its hydrophobicity and insolubility in water. Although the NC60 was estimated to have a great capacity to scavenge hydroxyl radicals by calculations of its Gibbs free energies of reactions for its dihydroxylations, it cannot fully exert its antioxidant activity, which is approximately 100-fold higher than that of ascorbic acid (AA) [21,22]. Techniques such as mechanochemical solubilization [23], solvent exchange [24], functional groups [25], and surface modification with solubilizing agents [26] have been used to stabilize aqueous C60 dispersions. Several surfactants enhanced the solubility of C60 including Tween 20, Tween 60, Tween 80, Triton X-100, polyvinylpyrrolidone, and polyoxyethylene lauryl ether [27,28]. They play an important role in preparing stable nanodispersions with enhanced bioavailability and efficacy by providing strong van der Waals or electrostatic forces. Notably, Pluronic polymer may be an excellent templating material for the dispersion of NC60 because of its inherent amphiphilic nature and generally recognized safety.

A biocompatible poly(ethylene oxide)–poly(propylene oxide) triblock copolymer, Pluronic, was employed to stabilize the carbon nanomaterials [29]. The safety of its use as a template or drug delivery system has been evaluated in several studies. Subsequently, various types of Pluronic polymers have been made available on the market with the approval of the United States Food and Drug Administration. Their characteristics can be tuned by varying the hydrophilic–lipophilic balance (HLB) and molecular weight. The HLB value is the fractional ratio of the lipophilic to hydrophilic regions of an amphiphilic molecule, indicating the hydrophilicity of a polymer. This is an important factor in determining the micellar behavior of Pluronic polymers. The hydrophobic poly(propylene oxide) moieties in Pluronic polymers exhibit strong affinity for the surfaces of hydrophobic carbon-based materials, while the hydrophilic poly(ethylene oxide) groups offer electrostatic repulsion in aqueous environments. The Pluronic polymers have been used to obtain a stable dispersion of C60 in a previous study [30]. However, to the best of our knowledge, there have been no reports on the evaluation and optimization of different types of Pluronic polymers as stabilizing agents of the NC60. Considering that the properties of polymers are important for determining the degree and quality of the NC60 dispersion, the Pluronic polymers with optimal HLB value and molecular weight may result in improved solubilization and dispersion of the NC60 in aqueous solutions for biomedical applications.

Therefore, in this study, different types of Pluronic polymers (F68, F127, L35, P123, and L81) were used as templates to develop Pluronic-coated NC60 nanoaggregates (NC60/Plu). The stability and solubilization properties of the NC60 were compared and optimized based on the HLB values and coating ratios of the Pluronic polymers. The physicochemical characteristics of NC60/Plu were analyzed using ultraviolet–visible (UV–Vis) spectroscopy and dynamic light scattering (DLS). The enhanced antioxidant activity of NC60/Plu was assessed both in situ and in vitro in MEFs.

## 2. Materials and Methods

### 2.1. Materials

Pluronic polymers (F68, F127, L35, P123, and L81), polyoxyethylene sorbitan monopalmitate (Tween 40), AA, C60, and tocopherol were purchased from Sigma-Aldrich (St. Louis, MO, USA). NC60 was purchased from DAEDAN Inc. (Incheon, Republic of Korea). The purity of NC60 was 92.72% without toxic heavy metals including Pb, As, Cd, Cr^2+^, and Hg. Chloroform, β-carotene, and linoleic acid were purchased from TCI (Tokyo, Japan). Hydrogen peroxide (H_2_O_2_, 30%) was purchased from Junsei Chemical Co. (Tokyo, Japan). NIH 3T3 murine fibroblast cells were obtained from the Korean Cell Line Bank (Seoul, Republic of Korea) for in vitro assays. Dulbecco’s modified Eagle medium (DMEM) and fetal bovine serum (FBS) were purchased from Gibco (Grand Island, NY, USA). Antibiotic-antimycotics were purchased from Thermo Fisher Scientific (Waltham, MA, USA). 2′,7′-Dichlorodihydrofluorescein diacetate (H_2_DCFDA) was purchased from Invitrogen (Carlsbad, CA, USA). Deionized (DI) water and phosphate-buffered saline (PBS) were obtained from HyClone (Logan, UT, USA). All the chemicals were used without further purification.

### 2.2. Preparation of Pluronic-Stabilized Natural Fullerene (NC60/Plu)

Different Pluronic polymers were used to prepare aqueous dispersions of NC60. Briefly, the Pluronic polymer (5, 25, 50, and 100 mg) was dissolved in 1 mL of DI water and mixed with 5 mg of NC60. The weight ratios of Pluronic polymers to NC60 were 1:1, 1:5, 1:10, and 1:20. The reaction was performed under magnetic stirring for 2 h at 300 rpm. Subsequently, 4 mL of DI water was added, and the mixture was stirred for another hour at 450 rpm. Subsequently, they were ultrasonicated for 4 h (amplitude = 20%, on/off time = 5 s/15 s). Unreacted NC60 was removed by centrifugation at 2000 rpm for 10 min. The resulting NC60/Plu is denoted by the type of Pluronic polymer used for stabilization (e.g., NC60/PF68 for NC60 coated with Pluronic F68). Bare NC60 was prepared using the same method in DI water without Pluronic polymer. As-prepared NC60 and NC60/Plu were freeze-dried for 3 days and stored at −20 °C until use. In detail, they were initially frozen in liquid nitrogen. Then, water from the frozen samples was removed by sublimation and desorption at −80 °C under a vacuum using a freeze dryer (FDU-8606, OPERON, Kimpo, Republic of Korea).

### 2.3. Characterization of NC60/Plu

Aqueous dispersions of NC60 and NC60/Plu were photographed for observation. UV–Vis absorption spectra were obtained in the wavelength range from 200 nm to 600 nm using rectangular quartz cuvettes by a UV–Vis spectrophotometer (Mega900; Scinco, Seoul, Republic of Korea). The samples were diluted in DI water (1:10) prior to measurement. The sample cell was reused and rinsed thoroughly in ethanol (70%) and dried under a stream of hot air between experiments. The intensity of the absorbance peak at 260 nm represents the amount of NC60 dispersed in the sample solution. Thus, it was applied to the equation below to calculate the yield of water-dispersible NC60.

(1) $$Yield\;\left(\%\right)=\frac{\left(Initial\;mass\;of\;NC60\right)-(remaining\;mass\;of\;NC60)}{\left(Initial\;mass\;of\;NC60\right)}\times 100$$

The hydrodynamic diameter, polydispersity index (PDI), and zeta potential of NC60 and NC60/Plu were determined in a fixed angle using a Zetasizer (ELSZ-2000; Otsuka, Osaka, Japan). The samples were diluted in DI water (1:10) prior to measurement. DLS analysis was carried out in disposable sample cells with a high-power semiconductor laser at a scattering angle of 90°. The zeta potential measurements were performed in a quartz sample cell supplied by the instrument manufacturer. The quartz cell was reused and rinsed thoroughly in DI water and dried under a stream of compressed air between experiments. The measurement temperature was kept at a constant room temperature of 25 °C. Characterization of NC60/PF127 containing different amounts of Pluronic was performed using the same techniques to determine the optimal coating ratio for NC60 stabilization.

### 2.4. Stability of NC60/Plu

The stability of NC60 and NC60/PF127 was evaluated by measuring hydrodynamic diameters and PDI after 4 weeks of storage at a constant room temperature of 25 ± 1 °C and humidity of 40 ± 2% using a Zetasizer. Partial aggregation of the unstable NC60 was observed and photographed during storage.

Lyophilization can critically affect the stability of nanoaggregates. Maintaining their physicochemical properties during lyophilization is crucial for the storage and transportation as a powder before use. Thus, any change in the characteristics of NC60/PF127 was monitored after lyophilization for successful application in the cosmeceutical and biomedical fields. We checked whether the freeze-dried NC60/PF127 powder was well dispersed in DI water without an increase in the hydrodynamic diameter and PDI.

### 2.5. In Situ Antioxidant Activity of NC60/Plu

The β-carotene bleaching assay was performed according to an optimally modified procedure [31,32]. Chloroform solutions of 44 μL of β-carotene (1.0 mg/mL; 8.2 mM), 17.6 µL of linoleic acid (0.1 mg/mL; 628 µM), and 88 μL of Tween 40 (0.2 mg/mL) were mixed. The solvent was removed in vacuo. The residual emulsion was immediately diluted with 9.6 mL of PBS (0.02 M, pH = 7.01) and dispensed 192 µL into a 96-well plate. Thereafter, antioxidant solutions containing AA, C60, NC60, or NC60/PF127 were prepared in DI water (0.5 µg/mL) and added to each well containing the diluted mixture. The final concentration of antioxidant samples was 0.02 µg/mL. The solution was mixed well and heated at 50 °C in a 96-well plate. The absorbance was measured every 5 min for 30 min using a microplate reader (SpectraMac ABS, Molecular devices, Seoul, Republic of Korea) to monitor the decrease in the absorbance of β-carotene at 460 nm.

### 2.6. In Vitro Cytotoxicity and Antioxidant Activity

For in vitro assay, NIH 3T3 mouse embryonic fibroblasts were plated at a density of approximately 5 × 10^4^ cells/mL in 90 mm cell culture dishes with DMEM supplemented with 10% FBS and 1% antibiotic-antimycotic solution. They were incubated in a humidified atmosphere of 5% CO_2_ at 37 °C and subcultured when cell densities reach 80% confluency. Then, the cells were collected by enzymatic digestion using 0.05% trypsin solution containing EDTA (Gibco, Waltham, MA, USA) before use as indicated in each experiment. The NC60/PF127 was used as a sample; however, NC60 was not suitable for in vitro testing because of its instability.

The cytotoxicity of NC60/PF127 was analyzed by monitoring the changes in cell viability after treatment with the nanoaggregates as described previously [33]. The cells were seeded in a 96-well plate at a cell density of 10,000 cells/well and stored for 12 h in an incubator with humidified atmosphere of 5% CO_2_ at 37 °C before sample treatment. Each test group was treated with NC60/PF127 at different concentrations of 0.01–1 mg/mL for 1 day. Control groups were treated with cell medium (0 mg/mL NC60/PF127). The Cell Counting Kit-8 (CCK-8) solution (Dojindo Laboratories, Kumamoto, Japan) was diluted in cell culture media (1:10) and added to a 96-well plate. Depending on the number of viable cells, a change in color to orange was observed, and the absorbance of the orange formazan was detectable using a microplate reader (VICTOR X3; PerkinElmer, Waltham, MA, USA) at a wavelength of 450 nm. The absorbance of each test group was compared with that of the control group to calculate cell viability using the following equation:

(2) $$Cell\;viability\;\left(\%\right)=\left(\frac{∆A_{450}\;of\;sample}{∆A_{450}\;of\;control}\right)\times 100$$

According to previous study, NIH 3T3 fibroblasts were stimulated with an oxidative stress agent, H_2_O_2_, to evaluate the antioxidant activity of NC60/PF127 [34]. In detail, the cells were seeded in a 96-well plate at a cell density of 10,000 cells/well and kept for 12 h in an incubator with humidified atmosphere of 5% CO_2_ at 37 °C before sample treatment. Then, the NC60/PF127 solutions at concentrations of 10–1000 ng/mL were treated simultaneously with H_2_O_2_ (5 μM) for 8 h. The ROS negative control was treated only with the cell medium, whereas the positive control was treated only with H_2_O_2_ solution. The cells were washed with PBS and incubated with a fluorescent probe for ROS, H_2_DCFDA for 90 min in the dark. The fluorescence intensities at 485 nm excitation and 528 nm emission wavelengths were measured using a microplate reader to calculate the in vitro ROS levels.

### 2.7. Statistical Analysis

Every experiment was performed in triplicate (*n* = 3) to obtain results expressed as average ± standard deviation. The significance of the differences between test groups was determined using Student’s *t*-test and one-way *ANOVA*. A *p*-value < 0.05 was considered statistically significant.

## 3. Results and Discussion

### 3.1. Preparation and Characterization of NC60/Plu

**Figure 1:**
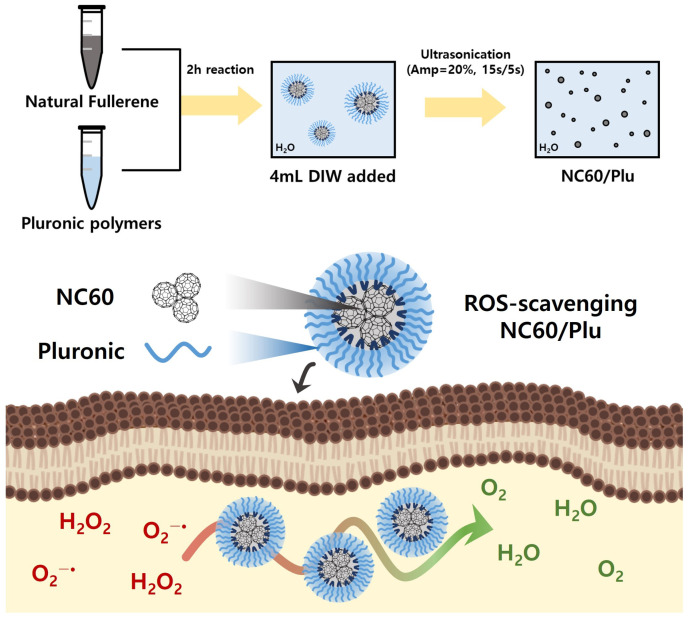
Schematic of the preparation and antioxidant activity demonstration of water-dispersible natural fullerene nanoaggregates using Pluronic polymers (NC60/Plu).

**Figure 2:**
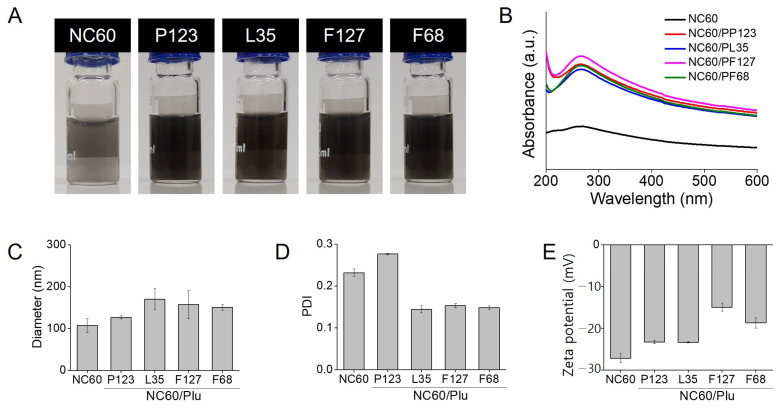
Characterization of natural fullerene (NC60) and Pluronic-coated NC60 nanoaggregates (NC60/Plu) with different hydrophile–lipophile balance values. (**A**) Photographs, (**B**) ultraviolet–visible (UV–Vis) absorption spectra, (**C**) hydrodynamic diameters, (**D**) polydispersity indexes (PDI), (**E**) zeta potentials of NC60 and NC60/Plu.

Water-dispersible NC60 was prepared via Pluronic templating and ultrasonication (Figure 1). A small amount of the NC60 can be dispersed only by ultrasonication; however, this requires a large amount of reagent and a long sonication time. Thus, amphiphilic Pluronic polymers were used as templates for the NC60, whereas PL81 with high lipophilicity was not suitable for preparing NC60/Plu. The hydrophilic and hydrophobic triblock structures of Pluronic were effective in coating the hydrophobic NC60. The synergistic effects of Pluronic templating and ultrasonication on NC60 solubilization are shown in Figure 2. The characteristic black color of the NC60 was very dim after insoluble NC60 was removed by centrifugation, whereas NC60/Plu maintained its black color (Figure 2A). The solubilization yield of NC60/Plu was approximately 2-fold higher than that of sonicated NC60 (23%). Pluronic polymers with different molecular weights and HLB values were used for NC60 stabilization, because they exhibit different micellization behaviors. The yields of NC60 solubilization based on the Pluronic type were 56% for NC60/PP123, 51% for NC60/PL35, 61% for NC60/PF127, and 56% for NC60/PF68. Accordingly, the UV–Vis absorption intensity at 260 nm was the highest for NC60/PF127 (Figure 2B). The hydrodynamic diameter of the NC60 increased from 107 to 170 nm after Pluronic coating (Figure 2C). Pluronic-templated nanoparticles with higher HLB values have larger hydrodynamic diameters owing to their more hydrophilic nature [35]. The PDI of NC60 and NC60/Plu were below 0.3 indicating a high homogeneity in particle size (Figure 2D). Owing to the neutral surface charge of Pluronic, the zeta potential of the NC60 shifted to a less positive value (Figure 2E). This shift increased as the solubilization yield increased, possibly because of the increased encapsulation of the NC60 by Pluronic. Therefore, PF127 was selected as the optimal templating material for NC60 solubilization.

**Figure 3:**
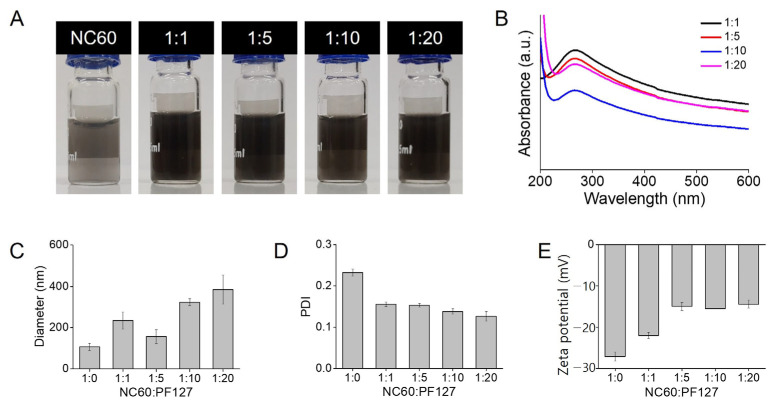
Characterization of natural fullerene (NC60) and Pluronic F127-coated NC60 nanoaggregates (NC60/PF127) at different polymer weight ratios (NC60:PF127 = 1:0, 1:1, 1:5, 1:10, and 1:20). (**A**) Photographs, (**B**) UV–Vis absorption spectra, (**C**) hydrodynamic diameters, (**D**) polydispersity indexes (PDI), (**E**) zeta potentials of NC60 and NC60/PF127 at different polymer weight ratios.

Varying weight ratios of PF127 were coated onto the NC60, resulting in a size-controllable NC60/PF127 (Figure 3A). Black dispersions of NC60/PF127 were successfully prepared at all weight ratios ranging from 1:1 to 1:20 (NC60:PF127). As shown in Figure 3B, the yield of NC60/PF127 was higher in the following order: 1:1 (65%), 1:5 (61%), 1:20 (58%), and 1:10 (43%). Despite the higher concentration of PF127, the solubilization yield of NC60/PF127 was lower, possibly because of its larger diameter and instability. Their hydrodynamic diameters increased with increasing PF127 concentration, resulting in an optimum 157 nm NC60/PF127 (Figure 3C). This phenomenon was also reported by Aich et al. [30]. The PDI of all nanoaggregates was below 0.3 (Figure 3D). The zeta potential of the NC60 shifted to neutral values as the amount of the PF127 coating increased (Figure 3E). Thus, NC60/PF127 (1:5), which exhibited a considerably high yield and the smallest diameter, was used in further experiments.

### 3.2. Stability of NC60/Plu

**Figure 4:**
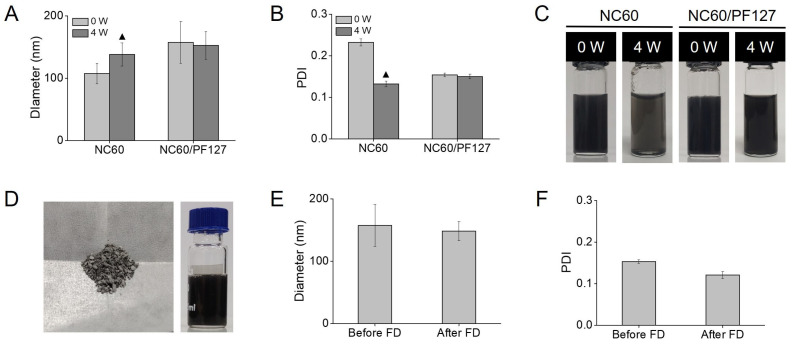
Stability analysis of natural fullerene (NC60) and Pluronic F127-coated NC60 nanoaggregates (NC60/PF127) at the polymer weight ratio of 1:5 in water at 25 °C. (**A**) Hydrodynamic diameters, (**B**) polydispersity indexes (PDIs), (**C**) photographs after 4 weeks of storage at 25 °C. ▲ denotes partial aggregation of the nanoaggregates. Characterization of NC60/PF127 after lyophilization (FD). (**D**) Photographs of powder (left) and aqueous dispersion of NC60/PF127 (right). (**E**) Hydrodynamic diameters and (**F**) PDIs of NC60/PF127 before and after FD.

The stabilities of the NC60 with and without the PF127 coating were compared after 4 weeks of storage at room temperature. Partial aggregation was observed in the aqueous NC60 dispersion, whereas NC60/PF127 was stable without any changes in the hydrodynamic diameter and PDI (Figure 4A,B). As shown in Figure 4C, the black color of the NC60 solution faded with the formation of aggregates at the bottom of the vial after 4 weeks. Meanwhile, the color of NC60/PF127 seldom changed, as the stability of the NC60 dispersion was enhanced by the Pluronic coating. PF127 has been used to improve the non-aggregating behavior of other all-carbon structures such as graphene and nanotubes [36,37]. This imparts electrostatic repulsion and prevents aggregation. Stable NC60/PF127 was lyophilized into a powder for ease of storage and transport without any challenges in its solubilization (Figure 4D). It maintained its diameter and PDI when redispersed in DI water (Figure 4D,F). Considering that many nanoaggregates necessitate cryoprotectants such as sucrose, glucose, mannitol, and trehalose to achieve successful lyophilization without irreversible aggregation, the NC/PF127 exhibited good stability during the freeze-drying process.

### 3.3. In Situ Antioxidant Activity of NC60/Plu

**Figure 5:**
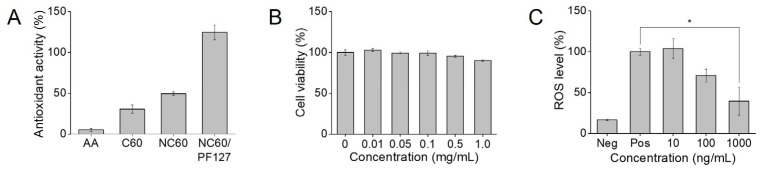
(**A**) In situ antioxidant activity of ascorbic acid (AA), fullerene (C60), natural fullerene (NC60), and Pluronic F127-coated NC60 nanoaggregates (NC60/PF127) via beta-carotene assay. In vitro cytotoxicity and antioxidant activity of Pluronic F127-coated NC60 (NC60/PF127) in CCK-8 and H_2_DCFDA assay. (**B**) Cell viability and (**C**) intracellular ROS level after treatment with NC60/PF127 (1:5). Negative control (Neg) and positive control (Pos) groups indicate the lowest and highest ROS levels, respectively (* *p* < 0.05).

The antioxidant activity of NC60 can be evaluated using many different methods. Previous studies have analyzed the capacity of NC60 to neutralize free radicals such as 2,2-diphenyl-1-picrylhydrazyl (DPPH), ABTS, peroxyl, and hydroxyl radicals [38,39]. Furthermore, the NC60 was used as an antioxidant to inhibit oxidation or protect against oxidative stress in the lipid peroxidation inhibition assay and the β-carotene bleaching assay [40]. The high degree of π-conjugation in both the NC60 and β-carotene allows for an accurate assessment of antioxidant activity compared to other methods such as DPPH and hydroxyl radical scavenging assays [41]. Therefore, the β-Carotene bleaching assay was performed to analyze the antioxidant activity of NC60 and NC60/PF127 (Figure 5A). AA and C60 were used as comparative samples. Treatment with antioxidants prevented the discoloration of β-carotene by inhibiting the attack of lipid radicals produced by linoleic acid oxidation. The retention of the yellowish color indicates antioxidant activity. The hydrophilic antioxidant, AA, could not restrain the fading of β-carotene at all. This result was consistent with that reported by Kato et al. [42]. Insoluble C60 and NC60 exhibited lower antioxidant activities (30.5% and 49.4%, respectively). As shown in Supplementary Figure S1, the NC60 had a better dispersity in water, resulting in stronger antioxidant activity in the β-Carotene bleaching assay. Water-dispersible NC60/PF127 had the strongest antioxidant activity, in which the color of β-carotene barely changed. The NC60 generally has strong ROS-scavenging activity due to its conjugated π-system, allowing effective neutralization of superoxide and hydroxyl radicals through electron transfer [43]. However, it is ineffective unless it is in a soluble form to scavenge free radicals [44]. Considering that the Pluronic alone does not have any antioxidant activity, it is suggested that polymer coating improves the antioxidant activity of the NC60 by increasing its accessibility and surface area exposed to ROS, enabling a faster encounter and interaction. Takada et al. reported the inhibitory activities of polyvinylpyrrolidone-entrapped and cyclodextrin-bicapped C60 [31]. Considering that the encapsulating materials do not have noticeable antioxidant activity, it was determined that the inhibitory effect of the antioxidant C60 was enhanced because of its solubilization and increased interactions with radicals.

### 3.4. In Vitro Cytotoxicity and Antioxidant Activity

Treatment of NC60/PF127 solutions at concentrations of 0–1 mg/mL did not alter the viability of the NIH 3T3 fibroblast cells (Figure 5B). There was a slight decrease in cell viability with an increase in the concentration of NC60/PF127. However, approximately 90% of the cells were alive after the addition of 1 mg/mL NC60/PF127. This suggests that NC60/PF127 is nontoxic to cells. Interestingly, the biological and environmental safety issues regarding aqueous C60 dispersions have long been debated. Several scientific reports called into question the usage of aqueous C60 dispersion [45,46,47]. Andrievsky et al. argued that C60 dispersions provoke negative biological effects because of impurities, such as organic solvent molecules and degradation products [48]. The authors noted that C60 is nontoxic and has positive effects on various biological activities. Accordingly, the water-dispersible NC60 prepared without using an organic solvent in this study did not cause severe cell death in NIH 3T3 fibroblasts. Furthermore, the Pluronic templating not only improves the dispersion stability of carbon materials but also their biocompatibility. The polymers may ameliorate the toxic effects of the NC60 by reducing its hydrophobicity and preventing its direct contact with biological molecules. This suggests that NC60/PF127 is safe for use in biomedical applications.

The C60 is well-known ROS scavenger that effectively inhibits ROS release. As shown in Figure 5C, NC60/PF127 scavenges the ROS produced by H_2_O_2_-stimulated cells in a concentration-dependent manner. The ROS level was decreased significantly to 39.5% after treatment of 1000 ng/mL NC60/PF127 (*p* < 0.05). The water-soluble NC60/PF127 can penetrate the cell membrane and reach the mitochondria, where ROS are mostly generated [49]. It may pass into the space between the inner and outer mitochondrial membranes containing an excess of protons that impart a positive charge to the surface of the nanoaggregates. This charge difference allows the NC60/PF127 NP to move across the inner mitochondrial membrane in order to reduce the generation of ROS. These results were consistent with those of other oxidative stress agents, such as lipopolysaccharide (LPS). Zhou et al. demonstrated that a liposomal formulation of amphiphilic C60 was capable of quenching the ROS produced by LPS [50]. In addition, the authors reported that the templating material enhanced the antioxidant activity of the C60 via its efficient delivery into cells. Other surfactant-coated C60 nanoparticles also exhibited beneficial effects on the prevention of cell death, reduction of chondrocyte apoptosis, and liver protection via ROS scavenging [51,52]. This finding implies that NC60/PF127 is a promising therapeutic agent for many ROS-mediated diseases.

## 4. Conclusions

Water-dispersible NC60 was successfully prepared by coating it with five types of Pluronic polymers. The yield of NC60 solubilization differed based on the HLB values and micellar behavior. It was revealed that PF127 is the most effective template for the NC60 in water. When the optimal weight ratio of PF127 was used to coat the NC60, the smallest NC60/PF127 ratio was obtained with a considerably high solubilization yield. NC60/PF127 exhibited the long dispersion stability and protection of β-carotene against the radical attack. Furthermore, in vitro assays determined the biocompatibility and prominent antioxidant activity of NC60/PF127, facilitating its application in the cosmetic and biomedical fields. In conclusion, we proposed the use of nature-extracted C60s as antioxidants via Pluronic templating. The NC60/PF127 effectively scavenged ROS and mitigated oxidative stress, ensuring its application in a wide range of conditions where oxidative damage is a central pathogenic factor. This makes NC60/PF127 an excellent therapeutic agent in managing and preventing ROS-associated diseases including neurodegenerative, cardiovascular, cancer, and inflammatory diseases.

## Data Availability

Data will be made available on request.

## Supplementary Materials

The following supporting information can be downloaded at: https://www.mdpi.com/article/10.3390/antiox13101240/s1, Figure S1: Photographs of fullerene (C60) and natural fullerene (NC60) dispersed in water.

## References

[1] Bosi S., Da Ros T., Spalluto G., Prato M. (2003). Fullerene derivatives: An attractive tool for biological applications. Eur. J. Med. Chem..

[2] Prato M. (1997). [60] Fullerene chemistry for materials science applications. J. Mater. Chem..

[3] Montellano A., Da Ros T., Bianco A., Prato M. (2011). Fullerene C_60_ as a multifunctional system for drug and gene delivery. Nanoscale.

[4] Brabec C.J., Gowrisanker S., Halls J.J.M., Laird D., Jia S.J., Williams S.P. (2010). Polymer-fullerene bulk-heterojunction solar cells. Adv. Mater..

[5] Yakuphanoglu F. (2008). Photovoltaic properties of the organic-inorganic photodiode based on polymer and fullerene blend for optical sensors. Sens. Actuator A.

[6] Niemann M.U., Srinivasan S.S., Phani A.R., Kumar A., Goswami D.Y., Stefanakos E.K. (2008). Nanomaterials for hydrogen storage applications: A review. J. Nanomater..

[7] Coq B., Planeix J.M., Brotons V. (1998). Fullerene-based materials as new support media in heterogeneous catalysis by metals. Appl. Catal. A.

[8] Mauter M.S., Elimelech M. (2008). Environmental applications of carbon-based nanomaterials. Environ. Sci. Technol..

[9] Kroto H.W., Heath J.R., O’Brien S.C., Curl R.F., Smalley R.E. (1985). C60: Buckminsterfullerene. Nature.

[10] Parthasarathy G., Srinivasan R., Vairamani M., Ravikumar K., Kunwar A.C. (1998). Occurrence of natural fullerenes in low grade metamorphosed Proterozoic shungite from Karelia, Russia. Geochim. Cosmochim. Acta.

[11] Reznikov V.A., Polekhovskiĭ Y.S. (2000). Amorphous shungite carbon: A natural medium for the formation of fullerenes. Tech. Phys. Lett..

[12] Kop T.J., Jakovljević D.M., Živković L.S., Žekić A., Beškoski V.P., Milić D.R., Gojgić-Cvijović G.D., Bjelaković M.S. (2020). Polysaccharide-fullerene supramolecular hybrids: Synthesis, characterization and antioxidant activity. Eur. Polym. J..

[13] Bolshakova O., Lebedev V., Mikhailova E., Zherebyateva O., Aznabaeva L., Burdakov V., Kulvelis Y., Yevlampieva N., Mironov A., Miroshnichenko I. (2023). Fullerenes on a nanodiamond platform demonstrate antibacterial activity with low cytotoxicity. Pharmaceutics.

[14] Galvan Y.P., Alperovich I., Zolotukhin P., Prazdnova E., Mazanko M., Belanova A., Chistyakov V. (2017). Fullerenes as anti-aging antioxidants. Curr. Aging Sci..

[15] Andrievsky G.V., Bruskov V.I., Tykhomyrov A.A., Gudkov S.V. (2009). Peculiarities of the antioxidant and radioprotective effects of hydrated C60 fullerene nanostuctures in vitro and in vivo. Free Radic. Biol. Med..

[16] Wang I.C., Tai L.A., Lee D.D., Kanakamma P.P., Shen C.K., Luh T.Y., Cheng C.H., Hwang K.C. (1999). C(60) and water-soluble fullerene derivatives as antioxidants against radical-initiated lipid peroxidation. J. Med. Chem..

[17] Cai X., Jia H., Liu Z., Hou B., Luo C., Feng Z., Li W., Liu J. (2008). Polyhydroxylated fullerene derivative C_60_(OH)_24_ prevents mitochondrial dysfunction and oxidative damage in an MPP^+^-induced cellular model of Parkinson’s disease. J. Neurosci. Res..

[18] Alfadda A.A., Sallam R.M. (2012). Reactive oxygen species in health and disease. J. Biomed. Biotechnol..

[19] Liu S., Chen D., Li X., Guan M., Zhou Y., Li L., Jia W., Zhou C., Shu C., Wang C. (2020). Fullerene nanoparticles: A promising candidate for the alleviation of silicosis-associated pulmonary inflammation. Nanoscale.

[20] Nozdrenko D., Matvienko T., Vygovska O., Bogutska K., Motuziuk O., Nurishchenko N., Prylutskyy Y., Scharff P., Ritter U. (2021). Protective effect of water-soluble C60 fullerene nanoparticles on the ischemia-reperfusion injury of the muscle soleus in rats. Int. J. Mol. Sci..

[21] Cheng X., Ni X., Wu R., Chong Y., Gao X., Ge C., Yin J.-J. (2018). Evaluation of the structure-activity relationship of carbon nanomaterials as antioxidants. Nanomedicine.

[22] Yoshitake Y., Michinobu T. (2024). Antioxidative activity of alcohol-soluble fullerene derivative. Fuller. Nanotub. Carbon Nanostruct..

[23] Bouchard D., Ma X., Isaacson C. (2009). Colloidal properties of aqueous fullerenes: Isoelectric points and aggregation kinetics of C60 and C60 derivatives. Environ. Sci. Technol..

[24] Deguchi S., Alargova R.G., Tsujii K. (2001). Stable dispersions of fullerenes, C_60_ and C_70_, in water. Preparation and characterization. Langmuir.

[25] Brettreich M., Hirsch A. (1998). A highly water-soluble dendro [60] fullerene. Tetrahedron Lett..

[26] Guldi D.M., Huie R.E., Neta P., Hungerbuhler H., Asmus K.D. (1994). Excitation of C60, solubilized in water by Triton X-100 and γ-cyclodextrin, and subsequent charge separation via reductive quenching. Chem. Phys. Lett..

[27] Torres V.M., Posa M., Srdjenovic B., Simplicío A.L. (2011). Solubilization of fullerene C60 in micellar solutions of different solubilizers. Colloids Surf. B Biointerfaces.

[28] Eom T., Barát V., Khan A., Stuparu M.C. (2021). Aggregation-free and high stability core-shell polymer nanoparticles with high fullerene loading capacity, variable fullerene type, and compatibility towards biological conditions. Chem. Sci..

[29] Lin Y., Alexandridis P. (2002). Temperature-dependent adsorption of pluronic F127 block copolymers onto carbon black particles dispersed in aqueous media. J. Phys. Chem. B.

[30] Aich N., Boateng L.K., Flora J.R.V., Saleh N.B. (2013). Preparation of non-aggregating aqueous fullerenes in highly saline solutions with a biocompatible non-ionic polymer. Nanotechnology.

[31] Takada H., Kokubo K., Matsubayashi K., Oshima T. (2006). Antioxidant activity of supramolecular water-soluble fullerenes evaluated by β-carotene bleaching assay. Biosci. Biotechnol. Biochem..

[32] Matsubayashi K., Goto T., Togaya K., Kokubo K., Oshima T. (2008). Effects of pin-up oxygen on [60]fullerene for enhanced antioxidant activity. Nanoscale Res. Lett..

[33] Li P., Han F., Cao W., Zhang G., Li J., Zhou J., Gong X., Turnbull G., Shu W., Xia L. (2020). Carbon quantum dots derived from lysine and arginine simultaneously scavenge bacteria and promote tissue repair. App. Mater. Today.

[34] Oh H., Lee J.S., Kim S., Lee J.-H., Shin Y.C., Choi W.I. (2023). Super-antioxidant vitamin A derivatives with improved stability and efficacy using skin-permeable chitosan nanocapsules. Antioxidants.

[35] Kim J.-Y., Choi W.I., Kim Y.H., Tae G., Lee S.-Y., Kim K., Kwon I.C. (2010). In-vivo tumor targeting of pluronic-based nano-carriers. J. Control. Release.

[36] Hong B.J., Compton O.C., An Z., Eryazici I., Nguyen S.T. (2012). Successful stabilization of graphene oxide in electrolyte solutions: Enhancement of biofunctionalization and cellular uptake. ACS Nano.

[37] Blanch A.J., Lenehan C.E., Quinton J.S. (2010). Optimizing surfactant concentrations for dispersion of single-walled carbon nanotubes in aqueous solution. J. Phys. Chem. B.

[38] Liu F., Xiong F., Fan Y., Li J., Wang H., Xing G., Yan F., Tai F., He R. (2016). Facile and scalable fabrication engineering of fullerenol nanoparticles by improved alkaline-oxidation approach and its antioxidant potential in maize. J. Nanopart. Res..

[39] Biswas R., Manley B.J., Xiao L., Siringan M.J., Crichton R.A., Stein J.B., Dawar A., Lee K.-B., Jin L., Li X. (2024). Reactive oxygen species scavenging capacity of functional fullerenes in solution and in macrophage cells. ACS Appl. Nano Mater..

[40] Grebowski J., Konopko A., Krokosz A., DiLabio G.A., Litwinienko G. (2020). Antioxidant activity of highly hydroxylated fullerene C_60_ and its interactions with the analogue of α-tocopherol. Free Radic. Biol. Med..

[41] Djordjecvic A., Canadanovic-Brunet J.M., Vojinovic-Miloradov M., Bogdanovic G. (2004). Antioxidant properties and hypothetic radical mechanism of fullerenol C_60_(OH)_24_. Oxid. Commun..

[42] Kato S., Aoshima H., Saitoh Y., Miwa N. (2009). Highly hydroxylated or γ-cyclodextrin-bicapped water soluble derivative of fullerene: The antioxidant ability assessed by electron spin resonance method and β-carotene bleaching assay. Bioorg. Med. Chem. Lett..

[43] Qiu Y., Wang Z., Owens A.C.E., Kulaots I., Chen Y., Kae A.B., Hurt R.H. (2014). Antioxidant chemistry of graphene-based materials and its role in oxidation protection technology. Nanoscale.

[44] Gharbi N., Pressac M., Hadchouel M., Szwarc H., Wilson S.R., Moussa F. (2005). [60]fullerene is a powerful antioxidant in vivo with no acute or subacute toxicity. Nano Lett..

[45] Sayes C.M., Fortner J.D., Guo W., Lyon D., Boyd A.M., Ausman K.D., Tao Y.J., Sitharaman B., Wilson L.J., Hughes J.B. (2004). The differential cytotoxicity of water-soluble fullerenes. Nano Lett..

[46] Oberdörster E. (2004). Manufactured nanomaterials (fullerenes, C60) induce oxidative stress in the brain of juvenile largemouth bass. Environ. Health Perspect..

[47] Zhu S., Oberdörster E., Haasch M.L. (2006). Toxicity of an engineered nanoparticle (fullerene, C60) in two aquatic species, Daphnia and fathead minnow. Mar. Environ. Res..

[48] Andrievsky G., Klochkov V., Derevyanchenko L. (2007). Is the C60 fullerene molecule toxic?!. Fuller. Nanotub. Carbon Nanostruct..

[49] Amani H., Habibey R., Hajmiresmail S.J., Latifi S., Pazoki-Toroudi H., Akhavan O. (2017). Antioxidant nanomaterials in advanced diagnoses and treatments of ischemia reperfusion injuries. J. Mater. Chem. B Mater. Biol. Med..

[50] Zhou Z., Lenk R.P., Dellinger A., Wilson S.R., Sadler R., Kepley C.L. (2010). Liposomal formulation of amphiphilic fullerene antioxidants. Bioconjug. Chem..

[51] Xiao L., Takada H., Gan X., Miwa N. (2006). The water-soluble fullerene derivative “Radical Sponge” exerts cytoprotective action against UVA irradiation but not visible-light-catalyzed cytotoxicity in human skin keratinocytes. Bioorg. Med. Chem. Lett..

[52] Yudoh K., Shishido K., Murayama H., Yano M., Matsubayashi K., Takada H., Nakamura H., Masuko K., Kato T., Nishioka K. (2007). Water-soluble C60 fullerene prevents degeneration of articular cartilage in osteoarthritis via down-regulation of chondrocyte catabolic activity and inhibition of cartilage degeneration during disease development. Arthritis Rheum..

