# Supplementary material for: Highly Water-Dispersed Natural Fullerenes Coated with Pluronic Polymers as Novel Nanoantioxidants for Enhanced Antioxidant Activity
Source: Antioxidants (Basel). 2024 Oct 15;13(10):1240. doi: 10.3390/antiox13101240 (PMC11505577; doi:10.3390/antiox13101240)
Supplement: Supplementary file 1 [file antioxidants-13-01240-s001.zip › antioxidants-3206034-supplementary.pdf]

Supporting Information

Highly water-disperse natural fullerenes coated with Pluronic polymers as novel nanoantioxidants for enhanced antioxidant activity

Hyeryeon Oh[a,b], Jin Sil Lee[a,b], Panmo Son[a,c], Jooyoung Sim[a], Min Hee Park[a], Young Eun Bang[d], Daekyung Sung[a], Jong-Min Lim[d,e,*], Won Il Choi[a,*]

[a] Center for Bio-Healthcare Materials, Bio-Convergence Materials R&D Division, Korea Institute of Ceramic Engineering and Technology, 202, Osongsaengmyeong 1-ro, Osong-eup, Heungdeok-gu, Cheongju, Chungbuk 28160, Republic of Korea

[b] School of Materials Science and Engineering, Gwangju Institute of Science and Technology, 123, Cheomdan-gwagiro, Buk-gu, Gwangju 61005, Republic of Korea

[c] Department of Applied Bioengineering, Graduate School of Convergence Science and Technology, Seoul National University, Seoul 08826, Republic of Korea

[d] Department of Electronic Materials, Devices, and Equipment Engineering, Soonchunhyang University, 22 Soonchunhyang-ro, Shinchang-myeon, Asan-si, Chungcheongnam-do 31538, Republic of Korea

[e] Department of Chemical Engineering, Soonchunhyang University, 22 Soonchunhyang-ro, Shinchang-myeon, Asan-si, Chungcheongnam-do 31538, Republic of Korea

*Corresponding authors

Won Il Choi

Tel: +82-43-913-1513

Fax: +82-43-913-1597

E-mail address: choi830509@kicet.re.kr (W.I. Choi)

Jong-Min Lim

Tel: +82-41-530-4961

Fax: +82-41-530-1717

E-mail: jmlim@sch.ac.kr (J.-M. Lim)

Keywords**:** fullerene; Pluronic; colloidal stability; antioxidant; ROS scavenging

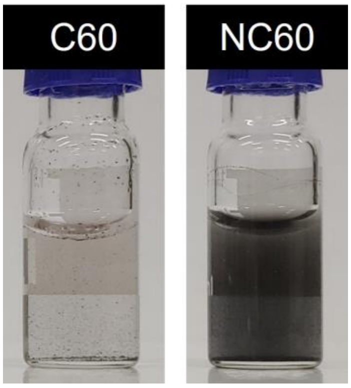

Figure S1. Photographs of fullerene (C60) and natural fullerene (NC60) dispersed in aqueous solution.
